# Soil microbial legacies influence plant survival and growth in mine reclamation

**DOI:** 10.1002/ece3.9473

**Published:** 2022-11-14

**Authors:** Katie McMahen, Shannon H. A. Guichon, C. D. Anglin, Les M. Lavkulich, Susan J. Grayston, Suzanne W. Simard

**Affiliations:** ^1^ Department of Forest and Conservation Sciences, Faculty of Forestry University of British Columbia Vancouver British Columbia Canada; ^2^ Anglin and Associates Consulting North Vancouver British Columbia Canada; ^3^ Faculty of Land and Food Systems University of British Columbia Vancouver British Columbia Canada

**Keywords:** ecological restoration, mycorrhizal fungi, plant–soil feedback, plant–microbe interactions, primary succession, root fungal community

## Abstract

Plants alter soil biological communities, generating ecosystem legacies that affect the performance of successive plants, influencing plant community assembly and successional trajectories. Yet, our understanding of how microbe‐mediated soil legacies influence plant establishment is limited for primary successional systems and forest ecosystems, particularly for ectomycorrhizal plants. In a two‐phase greenhouse experiment using primary successional mine reclamation materials with or without forest soil additions, we conditioned soil with an early successional shrub with low mycorrhizal dependence (willow, *Salix scouleriana*) and a later‐successional ectomycorrhizal conifer (spruce, *Picea engelmannii* × *glauca*). The same plant species and later‐successional plants (spruce and/or redcedar, *Thuja plicata*, a mid‐ to late‐successional arbuscular mycorrhizal conifer) were grown as legacy‐phase seedlings in conditioned soils and unconditioned control soils. Legacy effects were evaluated based on seedling survival and biomass, and the abundance and diversity of root fungal symbionts and pathogens. We found negative intraspecific (same‐species) soil legacies for willow associated with pathogen accumulation, but neutral to positive intraspecific legacies in spruce associated with increased mycorrhizal fungal colonization and diversity. Our findings support research showing that soil legacy effects vary with plant nutrient acquisition strategy, with plants with low mycorrhizal dependence experiencing negative feedbacks and ectomycorrhizal plants experiencing positive feedbacks. Soil legacy effects of willow on next‐stage successional species (spruce and redcedar) were negative, potentially due to allelopathy, while ectomycorrhizal spruce had neutral to negative legacy effects on arbuscular mycorrhizal redcedar, likely due to the trees not associating with compatible mycorrhizae. Thus, positive biological legacies may be limited to scenarios where mycorrhizal‐dependent plants grow in soil containing legacies of compatible mycorrhizae. We found that soil legacies influenced plant performance in mine reclamation materials with and without forest soil additions, indicating that initial restoration actions may potentially exert long‐term effects on plant community composition, even in primary successional soils with low microbial activity.

## Graphical abstract

Plants alter soil biological communities, generating ecosystem legacies that affect the performance of successive plants, influencing plant community assembly and successional trajectories. We found negative intraspecific (same‐species) soil legacies for an early successional shrub associated with pathogen accumulation, neutral to positive intraspecific soil legacies for a later‐successional conifer associated with ectomycorrhizal fungi, and neutral to negative soil legacies between conifers associating with different types of mycorrhizal fungi. Soil legacy effects of willow on next‐stage successional species (spruce and redcedar) were negative, potentially due to allelopathy, while ectomycorrhizal spruce had neutral to negative legacy effects on arbuscular mycorrhizal redcedar, likely due to the trees not associating with compatible mycorrhizae. Soil legacies influenced plant survival and growth in mine reclamation materials with and without forest soil additions, indicating that initial restoration actions have the potential to exert long‐term effects on plant community composition, even in primary successional soils with low microbial activity.

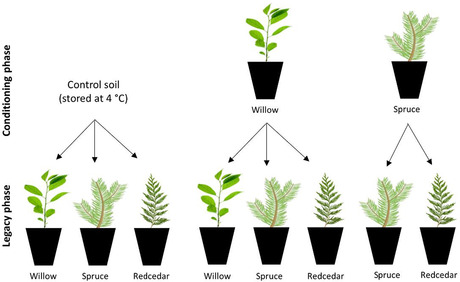

## 1 INTRODUCTION

Understanding the factors influencing plant establishment following severe disturbances is necessary for informing ecosystem restoration methods and enabling predictions of successional trajectories. The vegetation communities that develop during succession are a function of abiotic characteristics (e.g., soil properties, climate, topography), but also biological interactions (Connell & Slatyer, 1977). Plants shape soil physiochemical properties and soil biological communities through root development, root exudates, litter inputs, and creation of habitats for specific organisms (van der Putten et al., 2013). Changes in the soil environment based on plant identity leave “ecosystem legacies” that influence the fitness and niche space of successive plants, with effects on plant performance ranging from negative to positive (Bever et al., 1997; Ke & Wan, 2020).

Shifts in the abundances of soil organisms, including mutualists and pathogens, are increasingly recognized as drivers of ecosystem legacies. Legacies resulting from changes in the community composition of soil organisms can influence the structure and dynamics of plant communities through plant–soil feedbacks (Chung et al., 2019; Klironomos, 2002), and create historical contingencies in which the order and timing of past events affect community assembly during succession (Duhamel et al., 2019; Fukami, 2015). In the context of ecological restoration, this means that plant species selected for revegetation can create soil legacies that have the potential to influence the trajectory of the plant community.

Plant–soil feedbacks associated with soil biological legacies vary with plant nutrient acquisition strategy. In general, plant–soil feedbacks tend to be positive for ectomycorrhizal (EM) plants, neutral for arbuscular mycorrhizal (AM) plants, and negative for nitrogen‐fixing and non‐mycorrhizal plants (Teste et al., 2017). Variation in plant–soil feedbacks according to plants' nutrient acquisition strategies aligns with patterns in vegetation succession: later successional plants with greater mycorrhizal dependence tend to experience more positive feedbacks, while early successional plants, which commonly have more explorative root systems and low mycorrhizal dependence, tend to experience negative feedbacks (Cortois et al., 2016). Early successional plants typically invest more in growth than defense, making them susceptible to pathogens, which is accentuated by their lack of pathogen protection from mycorrhizal fungi (Koziol & Bever, 2015; Lemmermeyer et al., 2015). Accordingly, accumulation of soil‐borne pathogens can have negative legacy effects on early successional plants, breaking their dominance and promoting increases in plant diversity as well as species turnover in succession (Kardol et al., 2007; van der Putten et al., 1993).

As pathogen‐mediated negative soil legacies reduce the vitality and abundance of early successional plants, niche space opens for other plant species, including the next successional stage plant species. The subsequent plant communities that establish are a function of not only abiotic conditions and stochastic processes, such as dispersal, but also soil biological legacies (Dumbrell et al., 2010). In plant communities dominated by mycorrhizal‐dependent plants, particularly EM plants (associated with slower growth but increased pathogen resistance; Cheeke et al., 2019; Koziol & Bever, 2015), the buildup of mycorrhizal fungal propagules in the soil may generate positive legacies and promote establishment of plants that associate with those mycorrhizae. For example, Horton et al. (1999) found EM Douglas‐fir (*Pseudotsuga menziesii*) to establish near EM manzanita (*Arctostaphylos* spp.) but not AM chamise (*Adenostoma fasciculatum*). This is consistent with studies reporting overlap in the EM fungal taxa colonizing understorey and overstorey plants (Kennedy et al., 2003) and mid‐seral and late‐seral tree species (Kennedy et al., 2012). The presence of positive mycorrhizal‐mediated legacy effects in later successional plant communities is, however, ecosystem dependent; climax plant communities composed of AM and less mycorrhizal‐dependent plants, as opposed to EM plants, can be characterized by neutral to negative legacies, which prevent individual species from becoming dominant, promoting coexistence and diversity (Crawford et al., 2019; Mack et al., 2019).

Limited research has been conducted to understand if the soil legacy effects associated with plant nutrient acquisition type and successional stage described above are consistent under primary successional conditions, including mine reclamation settings (Zhu et al., 2022), and how the initial microbial community influences plant community assembly. Given the severely nutrient‐liming conditions in primary succession, simply growing plants in these barren soils can have positive legacies due to abiotic improvements in the soil, particularly when N‐fixing plants are present (Castle et al., 2016; Kuťáková et al., 2020; Png et al., 2019). In terms of biological legacies, neutral to negative biological legacies have been found for AM plants in early successional post‐glaciation soils (Castle et al., 2016) and foredunes (van der Putten et al., 1993), while Kuťáková et al. (2020) found soil fungi to potentially be positively linked to initial plant establishment in post‐mining soils, and Seeds and Bishop (2009) found positive legacies associated with N‐fixing bacteria in an area impacted by a volcanic eruption. These results suggest that while biological legacies in primary successional soils may be offset by abiotic legacies or weaker due to low abundances of soil organisms and low nutrient availability, they may be sufficiently present to influence plant community assembly. There is, however, a lack of studies in mine reclamation settings (Zhu et al., 2022) and on plants that dominate temperate forests, including conifers.

In a greenhouse experiment, we tested three hypotheses regarding how seedlings representing different functional groups and mycorrhizal associations create soil microbial legacies that influence the growth of successive plants in primary‐successional mine reclamation materials and mine reclamation materials amended with forest soil.

*H1*: Intraspecific (same‐species) legacy effects would vary with plant nutrient acquisition strategy and successional stage: willow (*Salix scouleriana* Barratt ex Hook.), an early successional shrub with low mycorrhizal dependence, would have negative intraspecific legacy effects corresponding to pathogen accumulation, while hybrid white spruce (*Picea engelmannii* Parry ex Engelm. × *glauca* (Moench)), a later successional stage EM conifer, would have positive intraspecific legacy effects associated with increased colonization by mycorrhizal fungi.

*H2*: Positive legacy effects would occur on next successional stage plants associating with compatible mycorrhizal fungal guilds, while a lack of compatible mycorrhizal fungal guilds would correspond with neutral legacy effects. Specifically, EM willow would have positive legacy effects on EM spruce, but EM willow and spruce would have neutral legacy effects on AM redcedar (*Thuja plicata* Donn ex D. Don).

*H3*: In unamended mine reclamation materials, which are low nutrient and have a lower abundance and diversity of soil organisms, legacy effects would be weaker than in mine reclamation materials amended with forest soil.

## 2 MATERIALS AND METHODS

### 2.1 Study system

Our greenhouse experiment used soils collected near the Mount Polley Mine, an open pit copper and gold mine in British Columbia (BC), Canada (52°32′42.35″ N, 121°37′58.67″ W). Glacial till subsoil (exposed by severe erosion) and deposited mine tailings were collected from the area disturbed by the 2014 tailings dam embankment failure (Figure S1, Table S1). The Mount Polley Mine tailings are non‐acid generating and have not shown negative toxicological effects on soil fauna or plants (Van Geest et al., 2017). Forest soil was collected from the adjacent, unimpacted Interior Cedar Hemlock (ICH) forest (Moist Cool subzone, Horsefly variant; Steen & Coupé, 1997) in mid‐ and late‐seral stands dominated by western redcedar, subalpine fir (*Abies lasiocarpa*), and hybrid white spruce (Figure S1, Table S1). At each forest site, litter was removed and the top 15–20 cm of soil collected, including the fermentation and humus layers, and the upper mineral horizons. Soils were homogenized and mixed with 20% v/v perlite to compensate for structural losses due to handling. This corresponds to a mixed soil sampling design as per Gundale et al. (2019), and thus the experiment evaluates the effects of the average or composite soil community of the sample locations. This design is suitable for mine reclamation research because in reclamation, soils commonly undergo some level of mixing during stockpiling and application. Physical and chemical properties of each soil type are presented in Table 1 (analytical methods are detailed in Appendix S1).

**TABLE 1:** Chemical and physical properties of forest soil, subsoil, and tailings collected from the Mount Polley Mine, British Columbia.

Parameter	Forest soil	Subsoil	Tailings
Sand (%)	44.46	48.12	62.07
Silt (%)	34.71	36.69	29.08
Clay (%)	20.83	15.18	8.85
Texture	Loam	Loam	Sandy loam
pH	5.2	8.09	8.23
EC	417	230	543
Total N (%)	0.605	0.025	0.013
Total C (%)	14.28	0.82	0.48
PO_4_‐P (mg kg^−1^)	41.98	1.42	1.21
CEC (cmol + kg^−1^)	35.14	8.93	6.74

*Note*: Forest soil includes the fermentation layer, humus layer, and upper mineral horizons.

Abbreviations: CEC, effective cation exchange capacity; EC, electrical conductivity.

### 2.2 Experimental design

**FIGURE 1:**
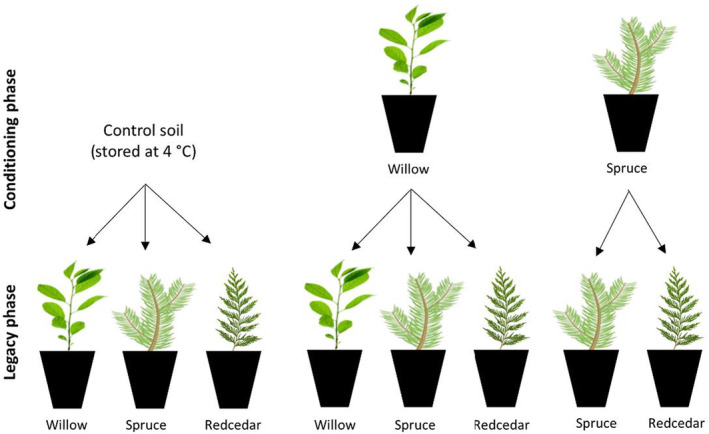
Combinations of conditioning‐ and legacy‐phase plant species tested in the two‐phase greenhouse experiment evaluating legacy effects on the same species and later‐successional species using willow (early successional), spruce (mid‐successional), and redcedar (mid‐ to late‐successional). The conditioning‐legacy combinations were carried out for three types of reclamation materials (tailings, subsoil, and tailings + subsoil) with or without forest soil additions (8 × 3 × 2 treatments).

In a two‐phase greenhouse experiment, plants species from different functional groups were grown to condition the soil microbiome (conditioning phase) followed by a second growing period (legacy phase) in which conditioning‐phase soil was used to grow the same plant species or a later‐successional‐stage plant species characteristic of ICH ecosystems. As shown in Figure 1, eight conditioning phase‐legacy phase combinations were tested (three unconditioned controls and five conditioned treatments). Soil conditioned by willow, an early successional EM shrub, was used to grow willow, hybrid white spruce (a mid‐successional EM tree), and western redcedar (a mid‐ to late‐successional AM tree). Spruce‐conditioned soil was used to grow spruce and redcedar. For comparison, in the legacy phase, control willow, spruce, and redcedar seedlings were grown in unconditioned soil that was stored at 4°C during the conditioning phase. Phospholipid fatty acidgh‐throughput Illumina MiSeq se analysis was conducted to verify that soil inoculum load did not decrease during the storage period (Appendix S2).

We applied two soil factors to all the conditioning‐legacy combinations, including the unconditioned controls: (1) three reclamation materials were tested (mine tailings, glacial till subsoil, and a 1:1 mixture by volume of tailings and subsoil, herein referred to as “tailings + subsoil”); and (2) reclamation materials were mixed with 25% v/v forest soil or left unamended. Three reclamation materials × two forest soil treatments × eight conditioning‐legacy combinations amounts to 56 treatments (Figure 1).

For both phases, five seeds collected from the Mount Polley Mine area were sterilized with hydrogen peroxide, sown into pots, and thinned to one seedling per pot. Containers were randomly arranged in the University of British Columbia (UBC) Horticulture Greenhouse, Vancouver, BC under 600 W high pressure sodium lights (17‐h photoperiod) and hand watered as needed. To mimic mine site conditions, seedlings were not fertilized during the experiment.

In the conditioning phase, seedlings were grown in 3 L pots in the greenhouse for 6 months, then harvested and the soil and seedlings retained. The roots of the conditioning‐phase seedlings were cut into approximately 1 cm pieces and mixed back into their respective soils. In the legacy phase, willow, spruce, and redcedar seedlings were grown in 250‐ml Ray Leach cones containing conditioned soil. Soil from each individual conditioning‐phase seedling formed an individual legacy‐phase treatment. Due to substantially higher mortality of willow seedlings than spruce seedlings in the conditioning phase, fewer replicates of legacy‐phase seedlings could be grown in willow‐conditioned soil treatments (*n* = 6) than in spruce‐conditioned and control soil treatments (*n* = 7).

### 2.3 Plant performance assessment

For each legacy‐phase seedling, initial germinant survival prior to thinning was recorded. Following harvest, shoots were dried for 72 h at 70°C and weighed. Samples of the youngest foliage were analyzed for total foliar nitrogen (N) and carbon (C) using an Elemetar vario EL Cube elemental analyzer at the UBC Stable Isotope Facility, Vancouver, BC. Washed spruce and redcedar roots were scanned using WinRhizo software to calculate specific root length (SRL; the ratio of root length to root dry biomass). Willow roots were not scanned to avoid inaccuracies associated with their dense, fibrous form. Root tips/segments for mycorrhizal fungal analysis were collected immediately postharvest and the remaining roots were dried for 72 h at 70°C then weighed.

### 2.4 Root fungal community assessment

For willow and redcedar, 40 × 1 cm randomly selected root segments per seedling were collected and frozen at −20°C until analysis. Twenty segments per sample were cleared in 10% KOH and stained with ink and vinegar (Vierheilig et al., 2005). Percent colonization of AM fungi and non‐AM fungal endophytes was evaluated using 100 intersections under a compound microscope as per McGonigle et al. (1990). For willow, EM fungal percent colonization was measured from the same stained roots using the gridline intersect method (Giovannetti & Mosse, 1980). To assess the identity and diversity of root‐associated fungi, DNA was extracted from the remaining 20 willow and redcedar root segments per sample using Qiagen DNeasy Plant Pro kits. High‐throughput Illumina MiSeq sequencing was conducted by Integrated Microbiome Resource at Dalhousie University, Halifax, Nova Scotia as per Comeau et al. (2017) using the fungal‐specific ribosomal RNA internal transcribed spacer (ITS) region ITS2 primers ITS86 and ITS4 (Turenne et al., 1999; Vancov & Keen, 2009; White et al., 1990). DNA characterization was only conducted for root samples from the 100% tailings and subsoil treatments, as well as 100% forest soil treatments (installed separately to support to support this simplified analysis), because the forest soil addition treatments represented a mixture of the analyzed soils, and therefore likely contained the same fungal species.

The resulting sequences were trimmed for quality using the sliding window function in Trimmomatic (version 0.39; Bolger et al., 2014) where the average quality per base within the four‐base‐pair window fell below fifteen. Sequences were then imported into QIIME2 (version 2019.7; Caporaso et al., 2010) and trimmed using Cutadapt to remove primers and read through (Martin, 2011). The ITS2 region was extracted and chimeras and non‐ITS2 sequences were removed using the ITSxpress QIIME2 plugin (version 1.8.0; Rivers et al., 2018). Bidirectional reads were assembled, denoised, and assigned to amplicon sequence variants (ASVs), dereplicated, and filtered for chimeras using the DADA2 pipeline (Callahan et al., 2016). Taxonomic identities of the ASVs were determined using a Naïve Bayes classifier trained on the UNITE database (Abarenkov et al., 2010). ASVs were assigned to fungal guilds using the FUNGuild database (Nguyen et al., 2016). Nonfungal sequences and singletons were removed, then data were rarified to the lowest number of reads.

For spruce, 50 random root tips were collected from each root system (or all tips for systems with <50 tips) and morphotyped under a dissecting microscope as per Goodman et al. (1996) to assess EM fungal percent colonization and diversity. For each morphotype, vouchers (one or a few root tips from an individual root system) were collected and stored at −20°C. Individual voucher specimens (minimum three per morphotype except for rare morphotypes observed less than three times) were ground with a sterile micropestle and sterile microsand followed by DNA extraction with PowerPlant Pro kits (Qiagen, 2017) as per the manufacturer's instructions. The ITS1 and ITS2 regions were amplified using polymerase chain reaction (PCR) with the fungal‐specific primers ITS4 (White et al., 1990) and ITS1F (Gardes & Bruns, 1993) using GE illustra PuReTaq Ready‐To‐Go PCR Beads, following the manufacturer's protocol. Thermocycler parameters for PCR involved an initial denaturation of 5 min at 94°C, followed by 35 cycles of denaturation at 94°C for 30 s, annealing at 55°C for 30 s, and elongation at 72°C for 30 s, followed by 7 min at 72°C for a final elongation. Bidirectional Sanger sequencing of PCR products was performed at Macrogen, Korea. Bidirectional reads were trimmed (error probability limit = 0.01) and aligned into consensus contigs using Geneious Prime (version 2019.2.3). The resulting sequences were identified using the Basic Local Alignment Search Tool (BLAST) against the National Center for Biotechnology Information (NCBI) GenBank database and the UNITE database (Abarenkov et al., 2010) with a 97% sequence similarity threshold.

Where morphotype voucher PCR products had more than one band in gel electrophoresis or Sanger sequencing failed, genomic DNA extracts underwent Illumina MiSeq sequencing and downstream bioinformatics as described above (except singletons were not removed and data were not rarified). Each morphotype voucher was assigned to the EM fungal taxon with the highest number of reads.

### 2.5 Statistical analysis

Statistical analyses were conducted in R (version 4.0.3; R Core Team, 2020). Linear models for each legacy‐phase plant species (willow, spruce, and redcedar) were constructed with soil treatment factors (reclamation material type × forest soil addition treatments × conditioning treatment (i.e., conditioning plant species)) as the explanatory variables with plant growth measures as the response variables. Response variables were natural log (log_*e*_) transformed as required to meet assumptions of normality and equal variance. Foliar N was included as a covariate to account for potential soil nutrient depletion during the conditioning phase. Depth to soil from the top of the pot was also included as a covariate to account for variation in soil settling and potential effects on light availability. Significance of model terms was tested with Type III ANOVA. Seedling survival and percent colonization by root symbionts were assessed using binomial generalized linear models (link = logit) with the same explanatory variables and covariates. Treatment effects were evaluated with pairwise post hoc contrasts using the *emmeans* function with the Tukey adjustment for multiple comparisons (Lenth, 2020). Regressions of shoot biomass against root symbiont colonization and SRL were used to evaluate relationships between response variables for reclamation materials with and without forest soil additions.

For willow and redcedar high‐throughput sequencing data, effects of conditioning treatment on root fungal community composition for each soil type were visualized using db‐RDAs conditioned on the covariates (foliar N and depth to soil) using the *capscale* function (*vegan* package; Oksanen et al., 2019). PERMANOVA significance testing was done with the *adonis* function in *vegan* (999 permutations). FUNGuild assignments were filtered to remove ASVs with multiple functional guild assignments, that is, fungi that have varying life histories (Nguyen et al., 2016). FUNGuild provides useful insight into potential shifts in microbial community structure; however, it is important to note that only 50% of willow ASVs and 42% of redcedar ASVs remained after filtering unassigned and multi‐guild fungi. Linear models of functional guild ASV richness and quasibinomial (link = logit) generalized linear models of functional guild ASV relative abundance were constructed with soil type × conditioning treatment as the explanatory variables along with the covariates described above. Post hoc pairwise testing with *emmeans* was used to test effects of conditioning treatments.

Indicator species analysis of spruce EM fungal morphotyping data as well as willow and redcedar high‐throughput sequencing data was done using the *multiplatt* function of the *indicspecies* package (de Caceres & Legendre, 2009; Nguyen et al., 2016). FUNGuild assignments were used to support interpretations (multi‐guild taxa were excluded from interpretations due to their unknown functional roles in the system).

## 3 RESULTS

Seedling responses were generally consistent among the reclamation materials tested (tailings, subsoil, and tailings + subsoil). Combined results across all reclamation material types are presented except where a significant effect of reclamation material type occurred.

### 3.1 Soil legacy effects on willow

**FIGURE 2:**
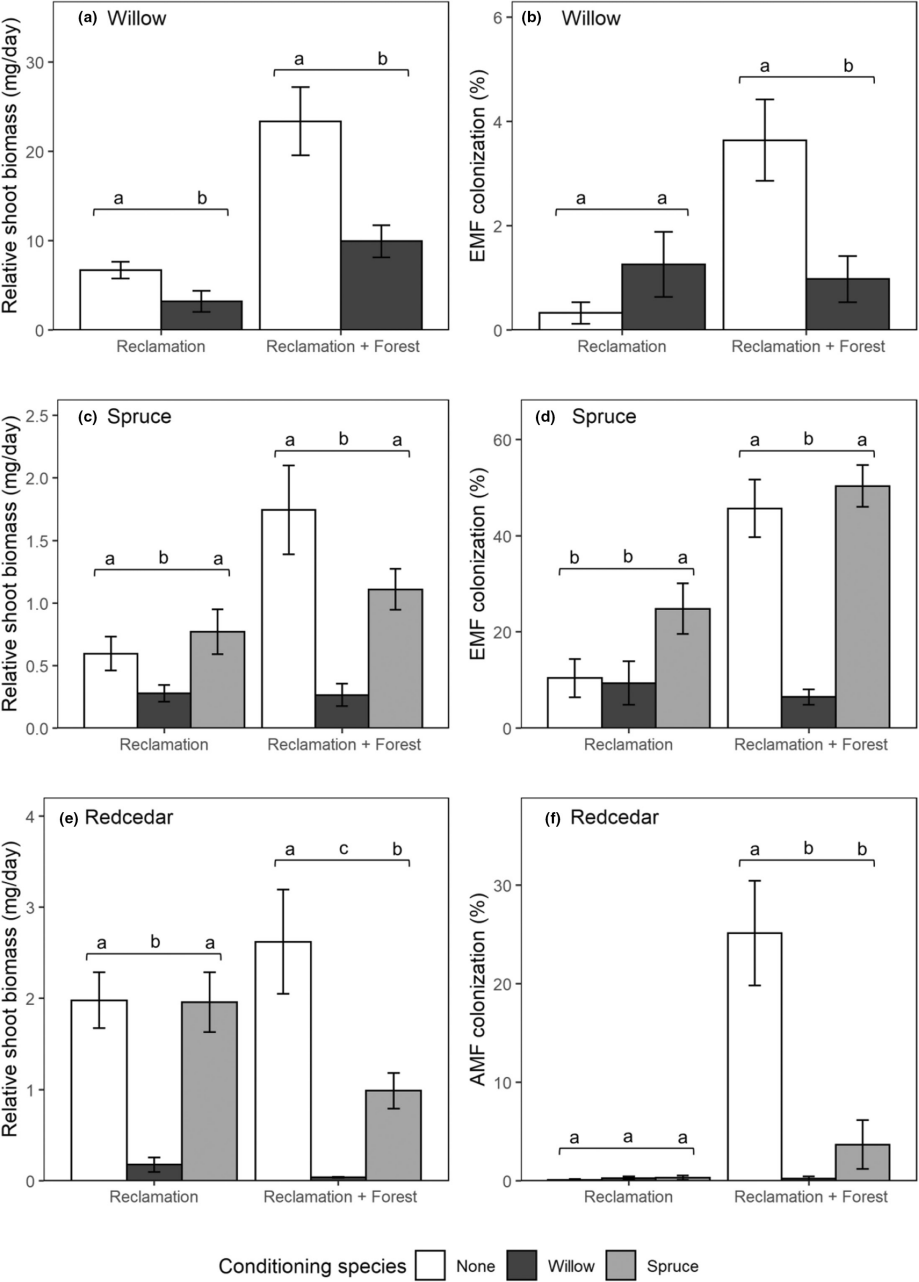
Shoot biomass (a, c, e) and percent root colonization by ectomycorrhizal fungi (EMF; b, d) and arbuscular mycorrhizal fungi (AMF; f) of willow (a, b), spruce (c, d), and redcedar (e, f) seedlings grown in control, willow‐conditioned, and spruce‐conditioned soils. Different letters indicate significant differences (*p* < 0.05) within each soil grouping: reclamation materials (tailings, subsoil, and tailings + subsoil) and reclamation materials mixed with 25% forest soil v/v.

**FIGURE 3:**
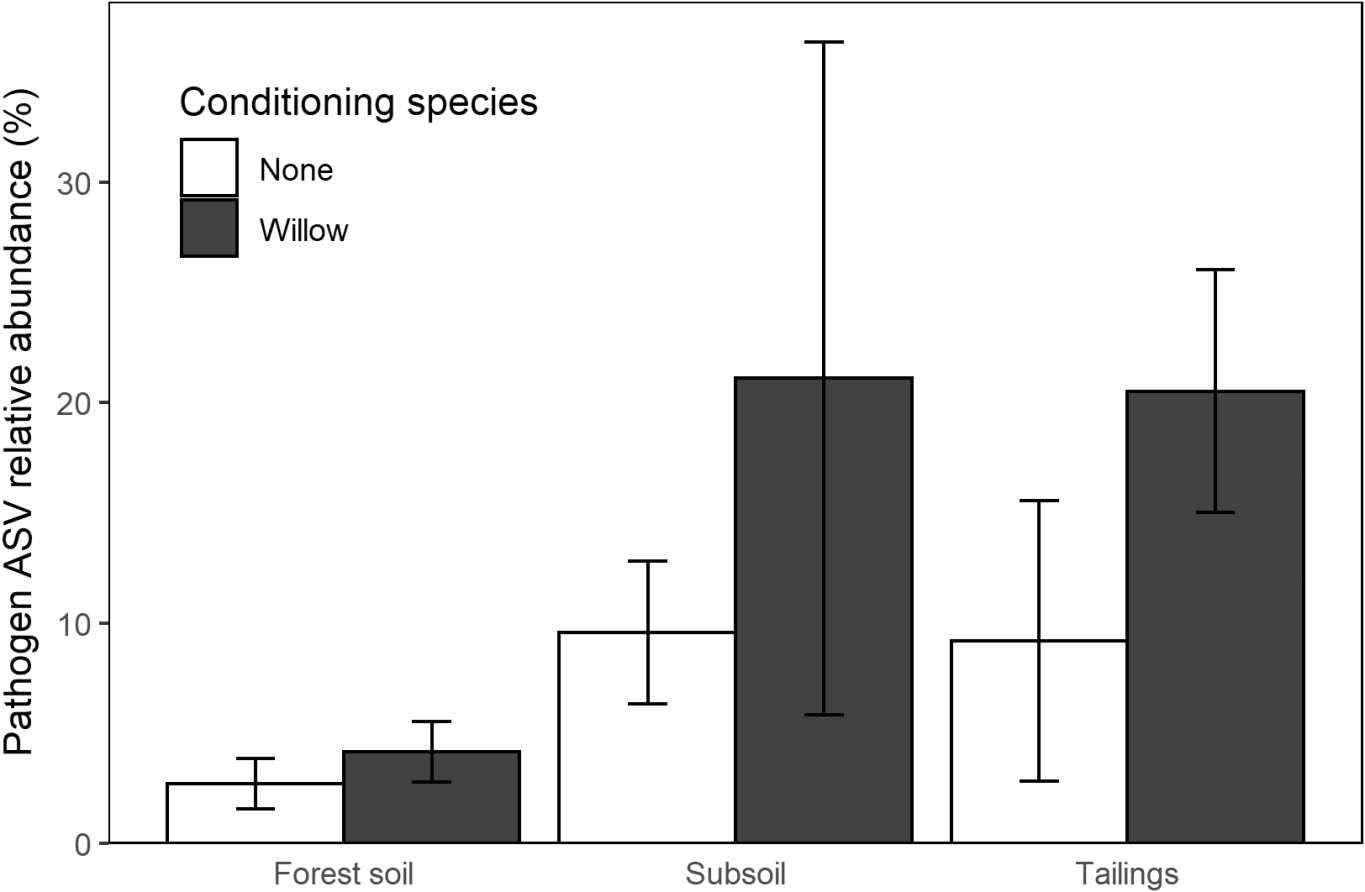
Mean willow root fungal pathogen amplicon sequence variant (ASV) relative abundance in forest soil (a), subsoil (b), and tailings (c) ± 1 SE. Pathogen relative abundance was greater in willow‐conditioned soils than control soils (*Z* = 2.36, *p* = .018) across all soil types.

Willow had lower germinant survival, shoot biomass, and root biomass in willow‐conditioned soils compared to control soils in reclamation materials with and without forest soil additions (*p* < .001; Figure 2, Table 2). High‐throughput sequencing of willow roots (forest soil, tailings, and subsoil treatments only) showed that fungal community composition was significantly different in willow‐conditioned treatments compared to controls for forest soil (PERMANOVA *R* ^2^ = .23, *p* = .001) and subsoil (PERMANOVA *R* ^2^ = .13, *p* = .043), and tended to be different for tailings (PERMANOVA *R* ^2^ = .18, *p* = 0.086; Figure S2). Relative abundance of ASVs of fungal pathogens was greater in willow‐conditioned soils compared to controls (*p* = .018; Figure 3). Fungal pathogen ASV richness tended to increase with willow‐conditioning in forest soil and tailings, although effects were not significant (*p* = .899; Figure S3). Indicator species analysis showed that plant fungal pathogens were maintained or gained in willow‐conditioned soil: *Plectosphaerella cucumerina* was dominant in control and willow‐conditioned subsoil and tailings (Table S2). Control forest soil was characterized by the pathogen *Neonectria candida*, while the pathogens *Moesziomyces aphidis* and *Plectosphaerella cucumerina* were indicative of willow‐conditioned forest soil.

**TABLE 2:** Mean ± standard error for each treatment group, with results combined across all reclamation materials (tailings, subsoil, and tailings + subsoil).

Legacy plant species	Forest soil addition	Conditioning plant species	Survival (%)	Relative shoot biomass (mg/day)	Relative root biomass (mg/day)	EM fungal colonization (%)	EM fungal richness	AM fungal colonization (%)	Fungal endophyte colonization (%)
Willow	No	None	88.6^a^ ± 3.1	0.9^a^ ± 6.7	2.0^a^ ± 0.3	0.3^a^ ± 0.2	–	0.0^a^ ± 0.0	23.7^a^ ± 3.3
		Willow	37.5^b^ ± 8.5	1.2^b^ ± 3.2	1.2^b^ ± 0.5	1.3^a^ ± 0.6	–	0.3^a^ ± 0.2	22.9^a^ ± 3.4
	Yes	None	97.4^a^ ± 1.7	3.8^a^ ± 23.4	8.9^a^ ± 1.3	3.6^a^ ± 0.8	–	0.0^a^ ± 0.0	35.5^a^ ± 2.3
		Willow	64.3^b^ ± 7.6	1.8^b^ ± 9.9	3.6^b^ ± 0.7	1.0^b^ ± 0.4	–	0.0^a^ ± 0.0	31.7^a^ ± 3.3
Spruce	No	None	86.6^ab^ ± 6.3	0.1^a^ ± 0.6	0.2^a^ ± <0.1	10.4^b^ ± 4.0	0.8^a^ ± 0.2	–	–
		Willow	65.4^a^ ± 12.1	0.1^b^ ± 0.3	0.1^b^ ± <0.1	9.4^b^ ± 4.6	0.7^a^ ± 0.2	–	–
		Spruce	94.3^b^ ± 3.3	0.2^a^ ± 0.8	0.2^a^ ± <0.1	24.8^a^ ± 5.2	1.0^a^ ± 0.2	–	–
	Yes	None	88.4^a^ ± 4.9	0.4^a^ ± 1.7	0.5^a^ ± 0.1	45.6^a^ ± 6.0	1.3^a^ ± 0.2	–	–
		Willow	81.5^a^ ± 8.2	0.1^b^ ± 0.3	0.1^b^ ± 0.0	6.5^b^ ± 1.6	0.9^b^ ± 0.2	–	–
		Spruce	92.1^a^ ± 5.6	0.2^a^ ± 1.1	0.4^a^ ± 0.1	50.3^a^ ± 4.4	2.0^a^ ± 0.1	–	–
Redcedar	No	None	87.1^a^ ± 5.6	0.3^a^ ± 2.0	0.7^a^ ± 0.1	–	–	0.1^a^ ± 0.1	22.6^a^ ± 3.4
		Willow	62.5^b^ ± 13.9	0.1^b^ ± 0.2	0.1^b^ ± 0.1	–	–	0.3^a^ ± 0.2	26.6^a^ ± 5.0
		Spruce	85.6^a^ ± 6.5	0.3^a^ ± 2.0	0.8^a^ ± 0.1	–	–	0.3^a^ ± 0.2	12.8^a^ ± 2.5
	Yes	None	87.5^a^ ± 5.8	0.6^a^ ± 2.6	1.0^a^ ± 0.2	–	–	25.1^a^ ± 5.3	37.2^a^ ± 3.3
		Willow	47.8^b^ ± 9.5	<0.1^c^ ± <0.1	0.0^c^ ± 0.0	–	–	0.2^b^ ± 0.2	21.4^a^ ± 3.2
		Spruce	82.4^a^ ± 7.0	0.2^b^ ± 1.0	0.4^b^ ± 0.1	–	–	3.7^b^ ± 2.5	27.7^a^ ± 2.6

*Note*: Different superscript letters denote significant differences in the results of the conditioning plant species treatments within each forest soil addition treatment group for each legacy plant species.

Fungal symbiont ASV richness decreased in the willow‐conditioned treatments compared to the controls for forest soil (*p* < .001) but not subsoil or tailings (Figure S3). The fungal symbionts *Cadophora finlandica* and *Cadophora* sp. were indicator species of control and willow‐conditioned forest soil, but *Tomentella sublilacina*, *Meliniomyces bicolor*, and *Phialocephala* sp. were indicative of control forest soil only (i.e., were not maintained in willow‐conditioned forest soil; Table S2). Willow root EM fungal colonization was low (≤5%) and there was no difference in percent colonization between willow‐conditioned and control soils for unamended reclamation materials (Figure 2). However, EM fungal colonization was lower in willow‐conditioned soils compared to control soils in reclamation materials with forest soil additions (Figure 2). There was no relationship between willow shoot biomass and EM fungal percent colonization in unamended reclamation materials (*R* ^2^_adj_ = .074, *p* = .599) or reclamation materials with forest soil additions (*R* ^2^_adj_ = .047, *p* = 1.00). Colonization of willow roots by AM fungi was low (≤2%) and did not differ among treatments (Table 2). Conditioning treatment had no significant effect on willow root percent colonization by non‐AM fungal endophytes (Table 2).

### 3.2 Soil legacy effects on spruce

Spruce seedling shoot and root biomass were lower in willow‐conditioned soils than control soils in reclamation materials with and without forest soil additions (*p* < .001; Figure 2, Table 2). A similar trend was observed for spruce germinant survival, which tended to be greater in control soils than willow‐conditioned soils (87% vs. 65% in unamended reclamation materials and 89% vs. 82% in reclamation materials with forest soil), although the effects were not significant (Table 2). There was no difference in shoot biomass, root biomass, and germinant survival of spruce seedlings between spruce‐conditioned soils and controls in reclamation materials with or without forest soil additions (Figure 2, Table 2).

Ectomycorrhizal fungal colonization of spruce roots was lower in willow‐conditioned soils than control soils for reclamation materials with forest soil additions (*p* < .001), but not for unamended reclamation materials (*p* = .169; Figure 2). EM morphotype richness was low (average richness ≤1.3) and did not differ between willow‐conditioned and control soils (Table 2). EM fungal colonization was greater in spruce‐conditioned soils than controls in unamended reclamation materials (*p* = .002; Figure 2), although a significant interaction with reclamation material type (*F*_4_ = 3.50, *p* = .003) showed this effect only occurred in subsoil and subsoil + tailings, not tailings. In reclamation materials with forest soil additions, EM morphotype richness (but not percent colonization) was greater in spruce‐conditioned soils than control soils (*p* < .001; Table 2). Consistent with EM fungal colonization and richness results, indicator species analysis of EM morphotypes showed that EM indicator species were lost in willow‐conditioned soil and maintained or gained in spruce‐conditioned soils (Tables S3 and S4). *Wilcoxina rehmii* was dominant in control soil and maintained in spruce‐conditioned soil, while *Thelephora terrestris* was characteristic of spruce‐conditioned soils only. Willow‐conditioned soil has no EM indicator species.

Across all conditioning treatments, there was a positive relationship between shoot biomass and EM fungal colonization in both unamended reclamation materials (*R* ^2^_adj_ = .091, *p* = .011) and reclamation materials with forest soil additions (*R* ^2^_adj_ = .225, *p* < .001). Additionally, there was a negative relationship between SRL and EM fungal colonization in reclamation materials with forest soil additions (*R* ^2^_adj_ = .389, *p* = .013), indicating that roots became thicker and less acquisitive when EM fungal colonization increased.

### 3.3 Soil legacy effects on redcedar

Redcedar shoot biomass, root biomass, and survival were lower in willow‐conditioned soils than control soils in reclamation materials with and without forest soil (Figure 2, Table 2). Redcedar shoot biomass, root biomass, and survival were also lower in spruce‐conditioned soils than control soils in reclamation materials with forest soil, but not in unamended reclamation materials (Figure 2, Table 2). Effects of conditioning treatment on the root fungal communities of redcedar seedlings were generally limited to soils with sufficient biological activity, that is, treatments containing forest soil amendments: AM fungal colonization of redcedar roots was lower in willow‐conditioned and spruce‐conditioned soils compared to controls in reclamation materials with forest soil additions, but there was no effect in unamended reclamation materials where AM fungal colonization was low (≤4%; Figure 2). Similarly, the fungal community of redcedar roots differed with conditioning treatment in forest soil (PERMANOVA *R* ^2^ = .40, *p* = .013) and subsoil (PERMANOVA *R* ^2^ = .35, *p* = .001), but not in tailings, which had the lowest biological activity (PERMANOVA *R* ^2^ = .19, *p* = .115; Figure S4).

Across all conditioning treatments, there was a positive relationship between shoot biomass and AM fungal colonization in reclamation materials with forest soil additions (*R* ^2^_adj_ = .438, *p* < .001), but not unamended reclamation materials (*R* ^2^_adj_ = −.003, *p* = .412). Additionally, there tended to be a negative relationship between SRL and AM fungal colonization in reclamation materials with forest soil additions (*R* ^2^_adj_ = .043, *p* = .072), indicating that roots became thicker and less acquisitive when AM fungal colonization increased.

Conditioning treatment had no significant effects on percent colonization of redcedar roots by non‐AMF fungal endophytes (Table 2). There were no significant changes in the relative abundance or species richness of fungal pathogens or non‐AMF symbionts in the high‐throughput sequencing results for redcedar roots. Indicator species analysis reflected this lack of clear shifts, as symbionts tended to be present in control and conditioned soil (*Cadophora luteo‐olivacea* in control and spruce‐conditioned tailings; *Cadophora* sp. in control, willow‐conditioned, and spruce‐conditioned forest soil; *Phalocephala* spp. in control and willow‐conditioned forest soil; Table S2). The only pathogenic indicator species was *Olpidium brassicae* in control subsoil and tailings, and it was not indicative of any conditioned soils (Table S2).

## 4 DISCUSSION

Understanding the processes underlying the recovery of vegetation communities in forest ecosystems following severe disturbances, such as mining, is critical for informing ecological restoration methods. This study showed that soil legacies mediated by soil microbes have the potential to influence plant community assembly and should be considered in ecosystem restoration and management.

### 4.1 Soil legacies promote plant species turnover and persistence

We found that willow, an early successional shrub with low mycorrhizal dependence, had negative intraspecific legacy effects associated with increased fungal pathogen abundance, while spruce, a later successional EM conifer, had neutral to positive intraspecific legacy effects associated with increased mycorrhizal fungal colonization and diversity. These findings support our first hypothesis that soil biological legacies would have contrasting effects depending on plant nutrient acquisition strategy, with non‐mycorrhizal plants (or plants with low mycorrhizal dependence) experiencing negative feedbacks and EM plants experiencing positive feedbacks (Teste et al., 2017). Our results align with results from studies in non‐forested ecosystems showing that plant–soil feedbacks associated with soil biological legacies shifted from promoting plant species turnover in early succession to promoting stability as succession progresses (Bauer et al., 2015; Kardol et al., 2006).

While we found evidence of root fungal pathogen accumulation corresponding to willow's negative intraspecific legacy effects, other soil biota, such as root‐feeding nematodes, may have also played a role as found by van der Putten et al. (1993) and Wilschut et al. (2019). Negative intraspecific legacy effects on willow also corresponded to decreases in EM fungal colonization and loss of EM fungal indicator species in reclamation materials amended with forest soil (but note in unamended reclamation materials where inoculum potential was low). Yet, there was no relationship between EM fungal colonization and willow shoot biomass, and mycorrhizal colonization rates on willow were low very low (≤ 5%), which supports pathogen accumulation as the dominant process. Our experimental design did not evaluate if pathogens were willow‐specific or generalists, but our results are consistent with early successional plants that have low mycorrhizal dependence and explorative root systems being sensitive to pathogens and experiencing negative legacy effects (Cheeke et al., 2019; Cortois et al., 2016; Koziol & Bever, 2015). Conversely, we found a positive relationship between spruce shoot biomass and EM fungal colonization. This is consistent with EM plants having greater mycorrhizal dependence and aligns with evidence that EM fungi can support tree recruitment (Seiwa et al., 2020) and persistence (McGuire, 2007) through positive soil biological legacies.

### 4.2 Mycorrhizal fungi generate legacies

In partial support of our second hypothesis, we found neutral to negative legacy effects of EM plants on AM redcedar, which does not associate with compatible mycorrhizal fungi. EM spruce had negative legacy effects on AM redcedar growth, but only in treatments containing forest soil (i.e., only where spruce had negative legacy effects on AM fungal colonization). Since redcedar trees were present in the forest soil collection areas and AM inoculum was in the forest soil, our results suggest that negative legacy effects on redcedar occurred due to the AM inoculum potential of the soil not being maintained by EM spruce and/or the conditioning‐phase conditions. In other scenarios where AM propagules are not initially present in the soil, effects on redcedar are expected to be neutral, as in the unamended reclamation materials. That AM fungi are drivers of the observed legacies is supported by the lack of spruce legacy effects on communities of fungal pathogens and non‐AM fungal endophytes on redcedar roots. These findings align with research showing established EM trees to have negative effects on AM seedling recruitment (Booth, 2004; Dickie et al., 2002; Guichon, 2015; Weber et al., 2005). Similarly, Seiwa et al. (2020) found hardwood recruitment following thinning may be limited to seedlings forming the same type of mycorrhiza. Thus, mycorrhizal fungal compatibility is likely an important factor in tree recruitment in forest ecosystems dominated by mycorrhizal‐dependent tree species.

Willow had negative legacy effects on spruce and redcedar seedling growth, which corresponded to negative legacy effects on mycorrhizal fungal colonization for both plant species, except in reclamation materials where inoculum potential was low. This was anticipated for AM redcedar because willow primarily associates with EM fungi, but no positive legacy effect on EM spruce contradicted our hypothesis. This may be due to the low mycorrhizal colonization observed in willow (i.e., willow and the conditioning‐phase conditions did not maintain the EM inoculum that was initially present in the forest soil) and/or low compatibility of willow and spruce EM fungal taxa, which highlights the importance of plant–mycorrhizal species compatibility within mycorrhizal fungal guilds (Ke & Wan, 2020). While most EM fungi are generalists, plant taxa‐specific EM fungi do occur (Massicotte et al., 1999) and a high proportion of host‐specific EM fungi have been reported to associate with early successional temperate forest plants (Twieg et al., 2007).

Negative willow legacy effects on spruce and redcedar growth also occurred in uninoculated reclamation materials where mycorrhizal fungal inoculum potential was low and no legacy effects on mycorrhizal fungal occurred, suggesting an additional legacy mechanism unrelated to root fungal communities was present. Nutrient depletion in the conditioning phase is an unlikely explanation, given that foliar N was incorporated into the models as a covariate (Cesarano et al., 2017). Accumulation of a generalist pathogen that was not assessed, such as an unclassified fungal taxon or root‐feeding nematodes, may have occurred. Additionally, willow produces salicylic acid, a phenolic compound implicated in plant defense responses. Salicylic acid has been shown to have allelopathic properties, reducing shoot growth in a variety of crop and weed species (Raskin, 1992). Allelopathy effects on next‐stage successional plant species may be a consequence of willow initiating defense responses to pathogen accumulation and/or an adaptation to delay willow's replacement in succession. We suspect that the negative legacy effects associated with willow may be plant species‐specific, especially if allelopathy is occurring, as other studies have found early successional plants to have neutral to positive legacies effects on co‐occurring plant species (Kuťáková et al., 2020; van de Voorde et al., 2011). This highlights the complexity of the interacting biotic and abiotic properties that generate soil legacies, and the potential for species‐specific inconsistencies with general ecological trends.

### 4.3 Legacy effects occur in primary successional soils

Soil legacy effects were generally consistent in mine reclamation materials and mine reclamation materials mixed with forest soil, leading us to reject our third hypothesis that legacy effects would be weaker or neutralized in primary successional conditions where biological colonization of soils is minimal. The only evidence supporting our third hypothesis was spruce seedling legacies had neutral effects on redcedar growth and AM fungal colonization in unamended reclamation materials (when AM presence was negligible), but negative effects in treatments containing forest soil. In contrast, the capacity of mycorrhizal fungi to contribute to soil legacies in primary succession is supported by our result of positive intraspecific legacy effects on spruce EM fungal colonization in reclamation materials. Our results suggest that where mycorrhizal fungi are a dominant mechanism for biotic legacies, negative legacy effects may be reduced or eliminated in barren primary successional soils (i.e., there is no inoculum to be “lost”), but positive legacies can be initiated despite the low abundance of mycorrhizal fungal propagules. This is consistent with studies in gravel quarry soils (Kuťáková et al., 2020) and areas impacted by volcanic eruptions (Nara, 2006; Seeds & Bishop, 2009) that have shown accumulation of microbial mutualists to support initial plant establishment in primary succession.

The negative intraspecific legacy effect we found for willow in unamended reclamation materials aligns with research showing that soil‐borne pathogen accumulation can still create negative legacies in primary successional soils, promoting turnover of early successional plants in foredune succession (van der Putten et al., 1993). Negative legacy effects of willow on spruce and redcedar in unamended reclamation materials indicate that the legacy mechanism, potentially accumulation of generalist pathogens or allelopathy, can occur irrespective of soil developmental phase. Our willow findings contrast with Kuťáková et al. (2020) and Castle et al. (2016), who found early successional plants to create neutral to positive legacies for co‐occurring plants in primary successional soils due to improvements in soil abiotic conditions (e.g., increasing nutrient levels). While our study focused on soil microbes and controlled for soil nutrient changes, results of the foliar nutrient covariate indicate that abiotic soil nutrient improvements may have occurred but did not shift net legacy effects. Variability in the results of studies in primary successional systems indicates that future studies should evaluate a broader scope of legacy mechanisms (e.g., allelopathy) to parse out the relative roles of biotic and abiotic mechanisms.

## 5 CONCLUSION

We found negative intraspecific soil legacies in willow, an early successional shrub with low mycorrhizal dependence, associated with pathogen accumulation, but neutral to positive intraspecific legacies in spruce, a later successional EM conifer, associated with increased mycorrhizal fungal colonization and diversity. Our findings support research showing that soil legacy effects vary with plant nutrient acquisition strategy, with non‐mycorrhizal plants experiencing negative feedbacks and EM plants experiencing positive feedbacks. Negative soil legacy effects of willow on next‐stage successional species (spruce and redcedar) were negative due to mechanisms not studied here, potentially allelopathy, while EM spruce had neutral to negative legacy effects on AM redcedar, likely due to the trees not associating with compatible mycorrhizal fungal. This suggests positive legacy effects mediated by soil microbes may be limited to scenarios where mycorrhizal‐dependent plants grow in soil containing legacies of compatible mycorrhizal fungal communities.

This study showed that soil legacies can influence plant survival and growth in mine reclamation materials both with and without forest soil additions, which suggests that initial restoration actions can exert effects on plant community composition (Wubs et al., 2019), even in primary successional soils with low microbial activity. This highlights the risks associated with “anything green is good” planting strategies or allowing invasive plants to establish (Dierks et al., 2019; Pickett et al., 2019), and supports restoration approaches that use native species to initiate natural successional trajectories. Consideration should also be given to revegetation with plants that build up communities of microbial mutualists that are relied upon by later successional plants. Our results indicate that revegetation plant selection in forest restoration can influence the successive plant communities that establish and show the importance of accounting for plant–microbe relationships in ecosystem restoration and management.

## AUTHOR CONTRIBUTIONS

**Katie McMahen:** Conceptualization (equal); data curation (lead); formal analysis (lead); funding acquisition (equal); investigation (lead); methodology (lead); project administration (lead); writing – original draft (lead); writing – review and editing (lead). **Shannon H. A. Guichon:** Data curation (supporting); supervision (supporting); writing – review and editing (supporting). **C. D. Anglin:** Supervision (supporting); writing – review and editing (supporting). **Les M Lavkulich:** Supervision (supporting); writing – review and editing (supporting). **Susan J. Grayston:** Supervision (supporting); writing – review and editing (supporting). **Suzanne Simard:** Conceptualization (equal); funding acquisition (equal); methodology (supporting); project administration (supporting); resources (lead); supervision (lead); writing – review and editing (supporting).

## Supporting information


Appendix S1

## Data Availability

High‐throughput sequencing data are openly available through GenBank (accession number PRJNA714120). Sanger sequencing data are openly available through GenBank (accession numbers are provided in Table S3). Experimental data are openly available in the Dryad Digital Repository (DOI: https://doi.org/10.5061/dryad.7h44j0zxz).
