# Supplementary material for: Soil microbial legacies influence plant survival and growth in mine reclamation
Source: Ecol Evol. 2022 Nov 14;12(11):e9473. doi: 10.1002/ece3.9473 (PMC9661428; doi:10.1002/ece3.9473)
Supplement: Supplementary file 1 — Appendix S1 [file ECE3-12-e9473-s001.docx]

# Supplementary Tables

**Table S1** Locations, dates and tree community compositions of the sites where tailings, subsoil and forest soil were collected at the Mount Polley Mine, British Columbia.

**Table S2** Indicator species analysis of willow and cedar root fungal communities from high-throughput sequencing for a subset of soil treatments (100% forest soil, subsoil and tailings only) and of spruce ectomycorrhizal fungi morphotypes (combined across all soil types). Symbiotic and pathogenic taxa are highlighted in green and red, respectively.

Table S2 cont’d

**Table S3** Sanger sequencing identification of ectomycorrhizal fungal taxa vouchers from morphotyping of spruce seedling roots.

**Table S4** High-throughput sequencing identification of spruce ectomycorrhizal (EM) fungal morphotype vouchers. For all but two vouchers analyzed with high-throughput sequencing, the amplicon sequence variant (ASV) with the most reads corresponded to an EM fungal sequence. Four vouchers contained mixed EM taxa (i.e., more than one dominant EM taxon), whereas the rest of the vouchers each had a single dominant EM fungal species present, but also had some saprophytic, parasitic and/or low abundance EM (0–4% of total reads) fungal DNA in the voucher specimen. Raw sequences data are available from GenBank (accession number PRJNA714120).

# Supplementary Figures


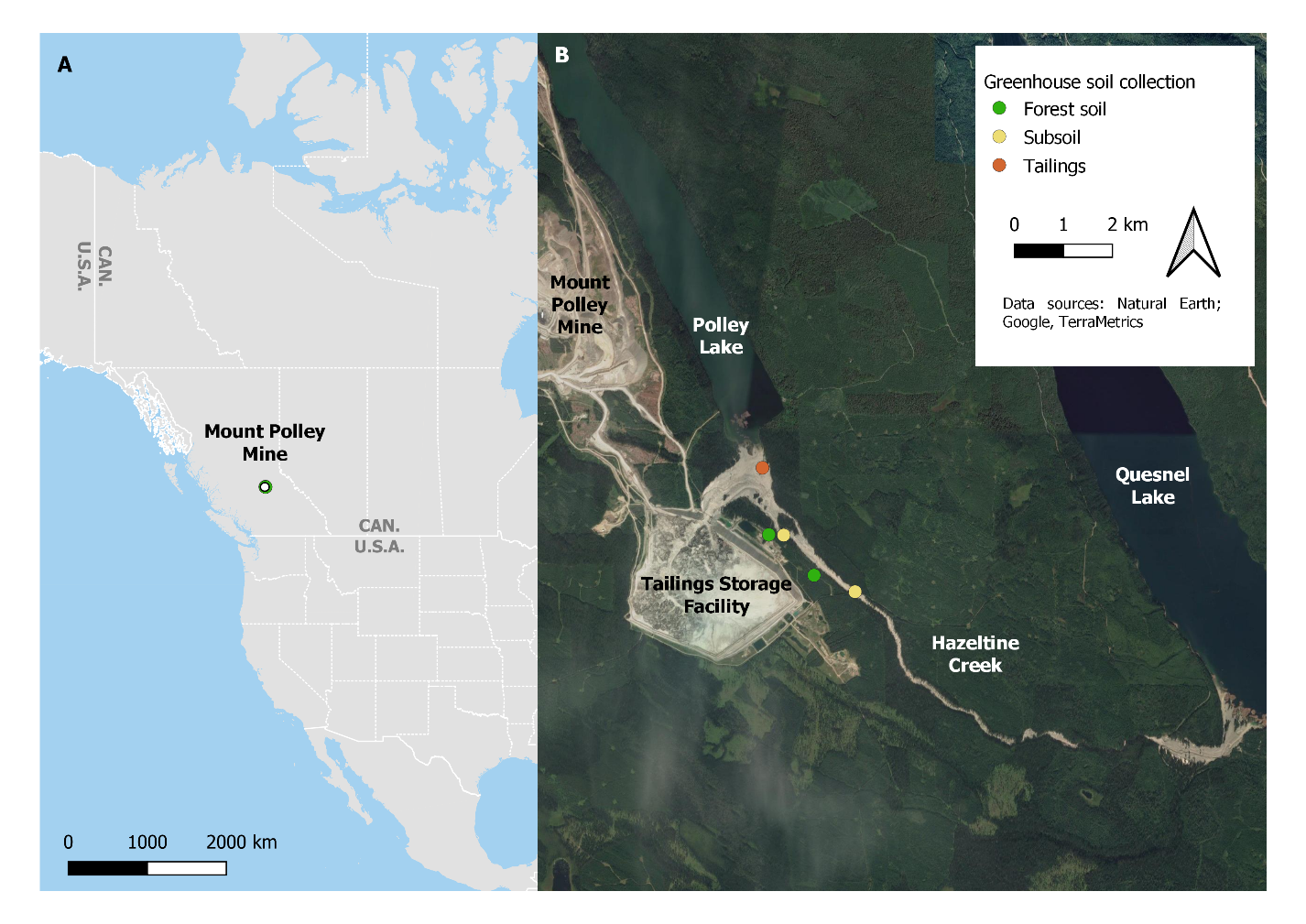


**Figure S1** Location of the Mount Polley Mine, British Columbia, Canada (a) where forest soil, subsoil (glacial till) and mine tailings were collected (b) for the greenhouse experiment.


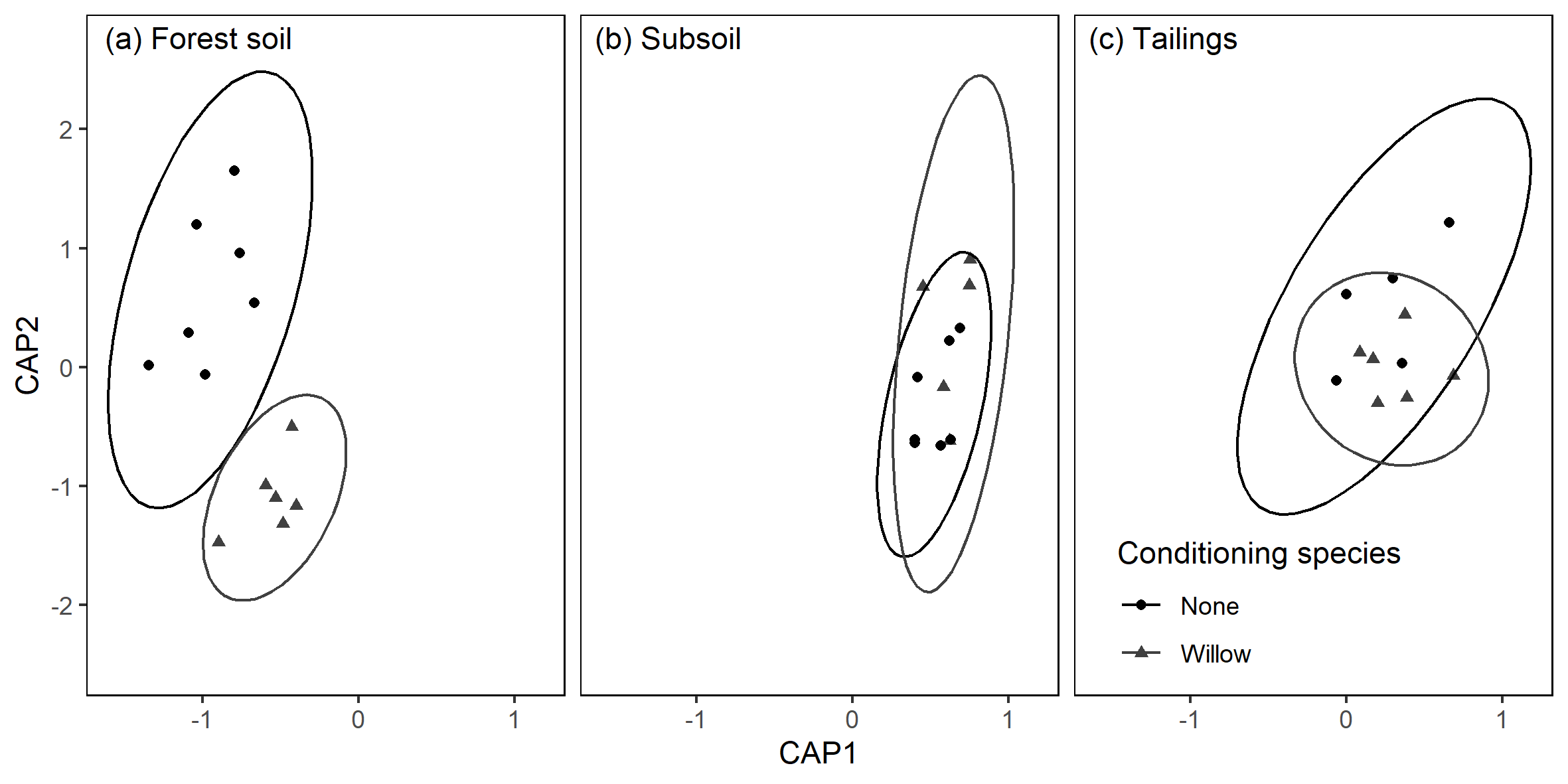


**Figure S2** db-RDA of willow root fungal communities (assessed through high-throughput sequencing) in willow-conditioned soils (forest soil, subsoil and tailings) compared to unconditioned control soils.


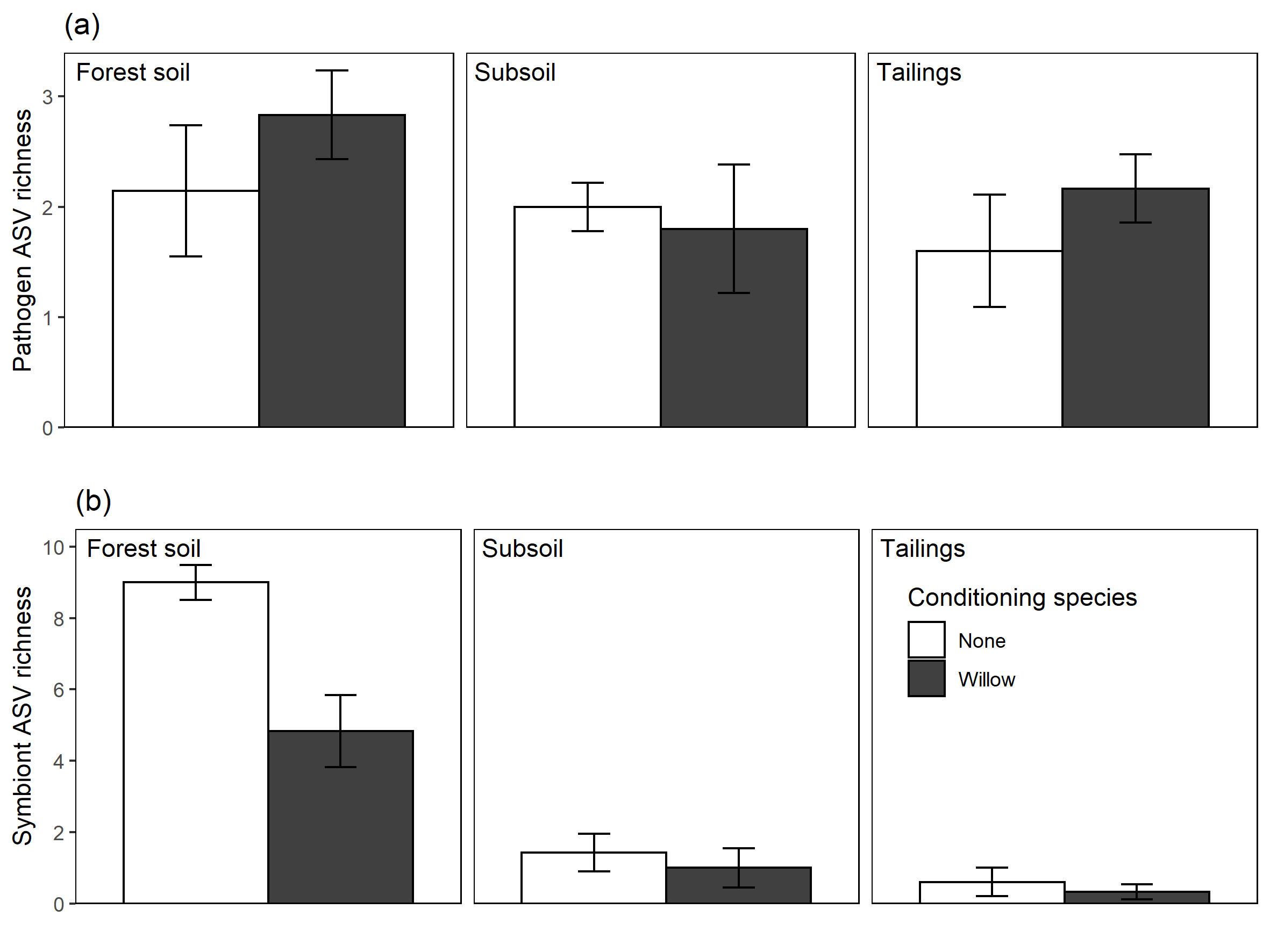


**Figure S3** Mean willow root fungal pathogen (a) and symbiont (b) amplicon sequence variant (ASV) richness in forest soil, subsoil and tailings ± 1 SE. Pathogen richness did not differ between willow-conditioned soils and control soils (*T*_28_ = 0.58, *P* = 0.899). Symbiont richness was lower in willow-conditioned soils compared to control soils (*T*_28_ = -4.14, *P* < 0.001).

**
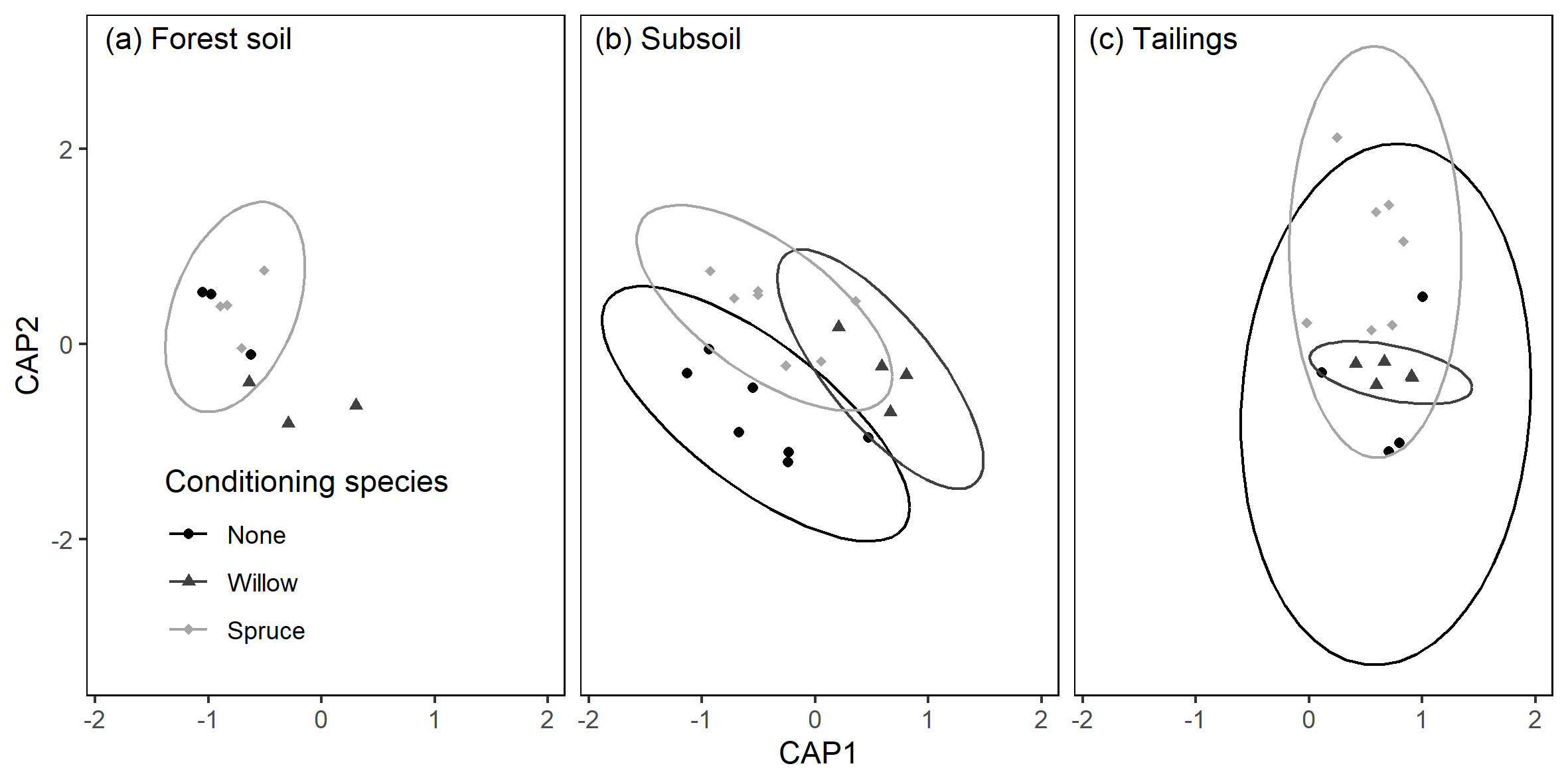
**

**Figure S4** db-RDA of redcedar root fungal communities (assessed through high-throughput sequencing) in willow- and spruce-conditioned soils (forest soil, subsoil and tailings) compared to unconditioned control soils. Ellipses could not be draw for willow and control (none) conditioning treatments in forest soil due to poor survival.

# Appendix S1. Soil physiochemical analyses

Soil physiochemical analyses were conducted to characterize the tailings, overburden and forest soil used in the greenhouse experiment. Soil samples were air dried and sieved to < 2 mm. pH and electrical conductivity (2:1 w/v deionized water:soil solution) were measured with a Seven Compact PH/Ion meter S220 and S230 Conductivity meter, respectively. The following parameters were analyzed by the British Columbia (BC) Analytical Laboratory, Victoria, BC: exchangeable cations (Al, Ca, Fe, K, Mg, Mn, Na) and effective cation exchange capacity through Barium chloride extraction (0.1 N) and ICP-OES analysis; total nitrogen and total carbon through combustion and analysis with an elemental analyzer; and available phosphorus (PO_4_-P; P) by Bray P-1 extraction (1-min shake) and UV-Vis analysis.

# Appendix S2. Phospholipid fatty acid analysis

Phospholipid fatty acid (PLFA) analysis was conducted to assess potential changes in the soil microbial community of the control soil stored at 4 °C during the conditioning phase. PLFAs were run on forest soil samples from each reference site (n = 3; Table S1) following soil collection and after the storage period. The storage period was twelve months due to: two months between initial collection and experiment initiation; having to re-start the conditioning phase after two months due to poor germination and survival; a six month growing period; and two months to process conditioning-phase seedlings, including morphotyping of EM fungi on spruce seedling roots. Comparisons of PLFA total and broad group microbial biomass from fresh and stored forest soil were done using linear models with storage treatment (baseline or stored) as the response variable.

PFLA results of stored forest soil samples indicated no inoculum load losses. Total PLFA biomass significantly increased (*P* = 0.016; Figure B-1). Similarly, biomass of all broad microbial groups increased (*P* < 0.05), yet, there were no significant differences in the proportion of each broad microbial group (Figure B-1). The only exception was protozoa where the proportion significantly increased (*P* < 0.001); however, protozoa make up < 1% of the overall microbial community.


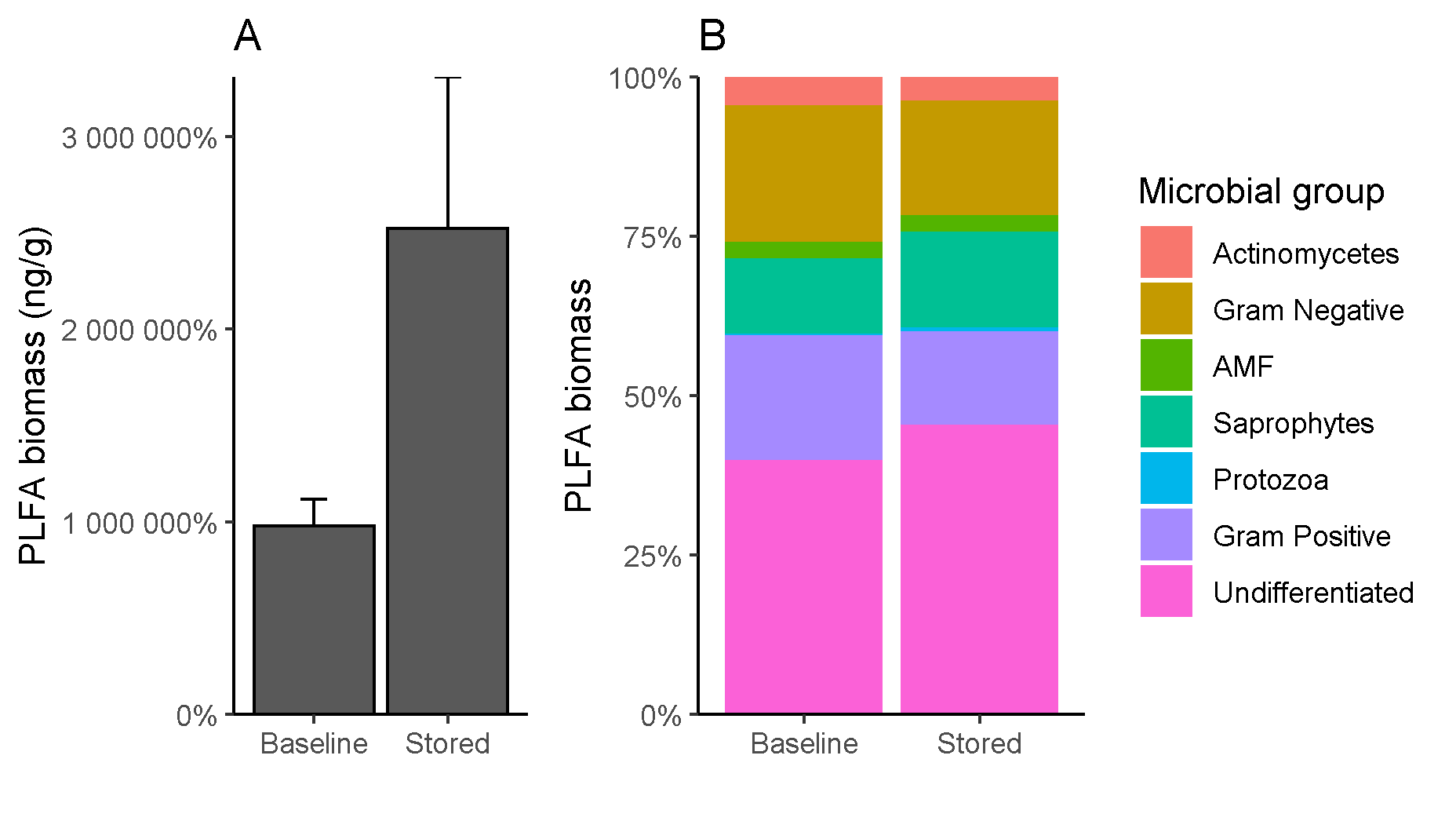


**Figure B-1** Phospholipid fatty acid (PLFA) total biomass (a) and microbial broad group biomass (b) results for control forest soil before (= Baseline) and after (= Stored) storage at -4 °C for the length of the conditioning phase of the greenhouse experiment.
